# Fusobacterium nucleatum as an Emerging Culprit in Brain Abscesses: A Narrative Synthesis of 25 Years of Clinical and Diagnostic Data

**DOI:** 10.7759/cureus.89421

**Published:** 2025-08-05

**Authors:** Fiza Ismail, Usama Mansoor, Haseeb Mehmood Qadri, Musharaf Zaman, Asim Ali, Muhammad Ans Asif, Muhammad Kaleem, Haysum Khan, Raahim A Bashir, Sundas Irshad, Asif Bashir

**Affiliations:** 1 Surgery, Lahore General Hospital, Lahore, PAK; 2 Neurological Surgery, Punjab Institute of Neurosciences, Lahore, PAK; 3 General Surgery, Lahore General Hospital, Lahore, PAK; 4 Anesthesia and Critical Care, Pakistan Red Crescent Teaching Hospital, Kasur, PAK; 5 General Surgery, Ghurki Trust Teaching Hospital, Lahore, PAK; 6 Radiology, Lahore General Hospital, Lahore, PAK; 7 Radiology, Woodlands Health, Woodlands, SGP; 8 Neurological Surgery, Shifa College of Medicine, Shifa Tameer-e-Millat University, Islamabad, PAK; 9 Neurological Surgery, State University of New York, New York, USA

**Keywords:** anaerobe, brain abscess, central nervous system infections, emerging rare pathogen, evidence, fusobacterium, gram negative, rare infection, review, suppuration

## Abstract

Brain abscesses are life-threatening infections, predominantly caused by anaerobic organisms. The role of oropharyngeal microbiota, presence in dental plaque biofilms, and hematogenous spread is established in the literature. However, due to its rare occurrence, limited literature is available on its management. The rationale of this narrative review was to elucidate risk factors, treatment strategies, and outcomes of brain abscesses caused by *Fusobacterium nucleatum* (*F. nucleatum*).

After a detailed literature search using PubMed, 12 cases were analyzed, containing a total of 13 patients, over the period of the last 24 years, between 2000 and 2024. All case reports and case series, including data relevant to brain abscess caused by *F. nucleatum*, were included, while letters to the editor and non-human studies were excluded. The main findings were male predominance (nine (69.23%)). The clinical presentations were headache (seven (53.85%)), fever (five (38.46%)), and neurological deficit (four (30.77%)), predominantly found in patients with poor oral health (seven (53.85%)), extracranial abscess sources with the presence of *F. nucleatum* (four (30.76%)), and alcohol consumption (four (30.76%)). Magnetic resonance imaging was the most commonly used radiological investigation in the majority of patients (nine (69.23%)). In biochemical investigations, blood cultures (eight (61.53%)) yielded negative results. However, brain abscess culture (four (30.76%)) as well as nucleic acid amplification test/polymerase chain reaction on culture-negative samples showed the presence of *F. nucleatum*. The majority of the treatment strategies were both surgical and medical. The surgical intervention involved craniotomy (three (23.07%)) and extraventricular drain placement, while medical management included courses of antibiotics such as metronidazole (eight (61.53%)), ceftriaxone (three (23.07%)), penicillin (three (23.07%)), and steroids (three (23.07%)). Injectable metronidazole 500 mg thrice daily was given for an average of 13 weeks, and injectable ceftriaxone 2 g once daily was given for an average of six weeks. Our results indicated that a combination of surgical and medical treatment leads to improvement in 10 (76.92%) patients. Follow-up MRI in four patients at an average of 23.4 weeks showed complete resolution in two (15.38%) patients and a reduction in abscess cavity size in the other two (15.38%).

This review underscores that both medical and surgical treatment together resulted in better outcomes for *F. nucleatum *brain abscesses and also advocates for oral examination in patients with brain abscesses, with prioritization of the identification of the primary source of infection.

## Introduction and background

Brain abscess is a focal collection of pus that can develop within the brain parenchyma and between the dura mater and arachnoid mater lining and contributes to significant mortality and long-term disability in patients. Mostly, brain abscess occurs due to a predisposing condition, pertaining to hematological spread from a sinus, ear infection, or due to complications following cranial surgery or trauma [1]. According to a systematic review published earlier, the most common causative organisms for brain abscesses were gram-positive organisms, with *Streptococcus* seen in 34% and *Staphylococcus* seen in 18% of patients. However, *Fusobacterium* species have also been identified in a minority of cases - 6% (7/98) of monomicrobial cases and 17% (4/24) in polymicrobial cases [2].

*Fusobacterium nucleatum* (*F. nucleatum*) is a gram-negative, spindle-shaped, non-spore forming, anaerobic bacterium and an important opportunistic pathogen. *F. nucleatum* is the most prominent gram-negative organism in dental plaque biofilms [3]. The opportunistic pathogen is also associated with various systemic diseases, including atherosclerotic cardiovascular diseases, inflammatory bowel disease, cancers (oral squamous cell carcinoma, colorectal cancer, breast cancer, genitourinary cancers), Alzheimer’s disease, rheumatoid arthritis, and respiratory diseases [4]. It is frequently encountered in extra-oral infections, such as those affecting the blood, brain, chest, lung, liver, joints, abdomen, as well as obstetrical and gynecological infections and abscesses [3]. Its association is often observed in advanced stages of disease states, chemo-resistance, and metastasis, and generally confers an unfavorable prognosis. Notably, *F. nucleatum* appears to act as a "facilitator" in cancer progression, particularly after non-cancerous cells transform into a malignant type [5].

In the context of cancer, colonization by *F. nucleatum* has been found to expedite tumor growth and metastatic progression [6]. There is limited research data available to guide optimal treatment modalities for brain abscess (i.e., surgical or medical management), with recommendations generally based on data and opinion from single-centers [1].

Keeping in view the rarity of brain abscess caused by *F. nucleatum*, the objective of this study was to compose an all-encompassing review aimed at determining the optimal approach for the detection and management of brain abscesses caused by *F. nucleatum*.

## Review

Methods

Literature Search Strategy

The data collection for this narrative review was performed using the database engine PubMed Central. The search was conducted using the Boolean operators ("AND" and "OR") with the following combination of keywords: “*Fusobacterium nucleatum* abscess”, “*Fusobacterium* brain abscess”, "*Fusobacterium nucleatum* intracranial abscess", "*Fusobacterium nucleatum* cerebral abscess", "*F. nucleatum* brain abscess", "*F. nucleatum* cerebral abscess", "*Fusobacterium* brain abscess", "*Fusobacterium*" AND "intracranial abscess", "*F. nucleatum*" AND "brain abscess", "brain abscess" AND "anaerobic bacteria" AND "*Fusobacterium*", "cerebral abscess" AND "*Fusobacterium*".

Eligibility Criteria

Study articles published from 2000 to 2024 were included in our narrative review. These were case reports and case series, studies on living humans, and studies that were in the English language. Exclusion criteria included letters to the editors, editorials, meta-analyses, cadaveric studies, and non-English studies.

Data Extraction

Four independent reviewers (FI, MZ, AA, MAA) screened, scrutinized all articles and extracted the data under the following variables: title, study type, signs and symptoms, site of lesion, radiological investigation such as computed tomography (CT) and magnetic resonance imaging (MRI), biochemical investigation such as nucleic acid amplification techniques (NAATs)/polymerase chain reaction (PCR), treatment, prognosis and follow-up. The data were then analyzed and verified for any errors, accuracy, and completeness. Data were entered into the pre-made Microsoft Office 365 tables (Microsoft® Corp., Redmond, WA) for stratification and analysis. Descriptive statistics were used to summarize the findings, and tables were generated to present the results in a structured manner.

Study Selection

A total of 300 articles were obtained as a result of the keyword terms in the databases. For this review, researchers used a literature search strategy outlined in Table 1.

**Table 1:** Summary of the literature search

Sr. no.	Search strategy	No. of publications
1.	Publications retrieved from the PubMed database	245
2.	Total number of publications from the electronic search	300
3.	Total number of publications after removal of duplication	190
4.	Total number of publications retrieved via keywords with available working links	98
5.	Publications remaining after the title or abstract screening	13
6.	Total included publications	12
7.	Total number of case reports	11
8.	Total number of case series	1

Results

In total, 11 case reports and one case series were included in this review. The majority of cases were reported from the United States of America (five), Germany (two), and one each from Belgium, Italy, France, the United Kingdom, and Taiwan (Table 2).

**Table 2:** Distribution of included studies by design and total number of patients

Sr. no.	Study type	Number of studies	Number of patients
1.	Case report	11	11
2.	Case series	1	2
Total		12	13

Most of the patients in these studies were male. One article did not specify the gender of the patient. The mean age was 48 ± 16 years (Table 3).

**Table 3:** Gender distribution of cases involved in the study, with a total of 13 patients, expressed as a percentage (%)

Sr. no.	Gender	Number of patients, n	Percentage occurrence % (n/N)
1.	Male	9	69.23%
2.	Female	3	23.07%

The most commonly reported presenting symptoms were headache (seven (53.85%)), fever (five (38.46%)), one-sided limb weakness (four (30.77%)), and confusion (four (30.77%)). Among patients with brain abscess, the most frequent physical findings were poor dental hygiene (seven (53.85%)) and hypertension (five (38.46%)) (Table 4).

**Table 4:** Symptoms distribution of cases involved in the study, with a total of 13 patients, expressed as a percentage (%)

Sr. no.	Presenting symptom	Number of cases, n (N = 13)	Percentage occurrence (n/N)
1.	Headache	7	53.85%
2.	Fever	5	38.46%
3.	Unilateral hemiparesis	4	30.77%
4.	Confusion/disoriented	4	30.77%
5.	Nausea/vomiting	2	15.38%
6.	Neck pain/stiffness	2	15.38%
7.	Lethargy	2	15.38%
8.	Generalized weakness	1	7.69%
9.	Periorbital pain	1	7.69%
10.	Blurred vision in the left eye	1	7.69%
11.	Abdominal pain	1	7.69%
12.	Diarrhea	1	7.69%
13.	Cough	1	7.69%
14.	Unstable gait	1	7.69%
15.	Dysarthria	1	7.69%

The most common investigation modality used in patients was MRI (nine (69.23%)), followed by CT scan (seven (53.85%)). Out of seven CT scans, two CTs were abdominal and chest, which revealed a hepatic abscess (two (15.38%)) and pulmonary nodules in the lung (one (7.69%)), respectively. Brain CT scan findings were consistent with space-occupying lesions (five (38.5%)), with perilesional edema, and ventriculitis (two (15.38%)) (Table 5).

**Table 5:** Radiological investigation used among patients involved in the study, with a total of 13 patients, expressed as a percentage (%) CT: computed tomography, CXR: chest X-ray, FDG-PET: fluorodeoxyglucose positron emission tomography, MRI: magnetic resonance imaging, USG: ultrasound

Sr. no.	Radiological investigations	Number of patients, n (N = 13)	Percentage occurrence (n/N)
1.	MRI	9	69.23%
2.	CT scan	7	53.85%
3.	Echocardiography	2	15.38%
4.	USG abdomen	1	7.69%
5.	FDG-PET	1	7.69%
6.	CXR	1	7.69%

The most common risk factors in patients with brain abscesses in these studies were poor dental hygiene (seven (53.85%)) and extracranial presence of *F. nucleatum* (four (30.76%)), followed by alcohol drinking (four (30.76%)) (Table 6).

**Table 6:** Risk factors of cases involved in the study, with a total of 13 patients, expressed as a percentage (%) COPD: chronic obstructive pulmonary disease, VSD: ventricular septal defect

Sr. no.	Risk factor	Number of cases, n (N = 13)	Percentage occurrence (n/N)
1.	Poor dental hygiene	7	53.85%
2.	Presence of *Fusobacterium nucleatum* in extracranial abscess and oral cavity	4	30.76%
3.	Alcohol drinking	4	30.76%
4.	Smoking	3	23.07%
5.	Hypertension	2	15.38%
6.	Endocardial vegetations	2	15.38%
7.	Atrial fibrillation	1	7.69%
8.	Mitral valve prolapse	1	7.69%
9.	Cardiomyopathy	1	7.69%
10.	VSD	1	7.69%
11.	COPD	1	7.69%
12.	Betel chewing	1	7.69%
13.	Illicit drug use (cannabis, amphetamines)	1	7.69%

Most of the cerebrospinal fluid (CSF) findings were consistent with bacterial infection, and the common sites of lesion in patients with brain abscesses in these studies were frontal (four (30.76%)) and cerebellar lobes (four (30.76%)), with an average lesion size of 2.15 ± 0.91 cm (Table 7). Most of the lesions were left-sided (six (46.15%)), followed by right-sided (five (38.46%)), and bilateral (three (23.07%)).

**Table 7:** Most common site of lesion among patients involved in the study, with a total of 13 patients, expressed as a percentage (%)

Sr. no.	Site of lesion	Number of cases, n (N = 13)	Percentage occurrence (n/N)
1.	Frontal	4	30.76%
2.	Cerebellar	4	30.76%
3.	Temporal	2	15.38%
4.	Occipital	2	15.38%
5.	Parietal	2	15.38%
6.	Parieto-temporo-occipital junction	1	7.69%

The most common diagnostic intervention yielding positive results for *F. nucleatum* in these patients was the nucleic acid amplification test (NAAT) (nine (69.23%)), followed by abscess culture (six (46.15%)) and gram staining (two (15.38%)) (Table 8).

**Table 8:** Diagnostic modality of the cases involved in the study, with a total of 13 patients, expressed as a percentage (%) NAAT: nucleic acid amplification test

Sr. no.	Intervention	Number of cases, n (N = 13)	Percentage occurrence (n/N)
1.	NAAT	9	69.23%
2.	Abscess culture	6	46.15%
3.	Gram staining	2	15.38%

The cultures were obtained from CSF, brain abscesses, and liver abscesses of affected patients. The majority of samples showed growth of *F. nucleatum*, mostly for patients with brain abscess (four (30.77%)). However, the most frequent negative results for *F. nucleatum* in these studies were observed in blood cultures (eight (61.53%)) (Tables 9-10).

**Table 9:** Positive cultures showing growth of Fusobacterium nucleatum among cases involved in the study, with a total of 13 patients, expressed as a percentage (%) CSF: cerebrospinal fluid

Sr. no.	Positive culture	Number of cases, n (N = 13)	Percentage occurrence (n/N)
1.	Brain abscess	4	30.77%
2.	Liver abscess	2	15.38%
3.	CSF	1	7.69%

**Table 10:** Type of cultures yielded no growth of Fusobacterium nucleatum among cases involved in the study, with a total of 13 patients, expressed as a percentage (%) CSF: cerebrospinal fluid

Sr. no.	Negative culture	Number of cases, n (N = 13)	Percentage occurrence (n/N)
1.	Blood culture	8	61.53%
2.	CSF culture	6	46.15%
3.	Brain abscess	1	7.69%

Among the samples on which NAAT/PCR was performed, brain abscesses (five (38.46%)) and CSF (four (30.77%)) were the most common sources showing presence of *F. nucleatum* (Table 11).

**Table 11:** Distribution of sample showing presence of Fusobacterium nucleatum in performing nucleic amplification technique, involved in the study, with a total of 13 patients, expressed as a percentage (%) CSF: cerebrospinal fluid, NAAT: nucleic acid amplification test, PCR: polymerase chain reaction

Sr. no.	NAAT	Number of cases, n (N = 13)	Percentage occurrence (n/N)
1.	Brain abscess	5	38.46%
2.	CSF	4	30.77%
3.	Oral cavity PCR	2	15.38%
4.	Pulmonary abscess	1	7.69%

The CSF cytology was suggestive of bacterial infection in (six (46.15%)) of cases, and the microscopic examination of the brain abscess showed neutrophilic infiltration (two (15.38%)), abscessed cavity (two (15.38%)), necrosis (two (15.38%)), as well as granulation tissue (two (15.38%)), macrophage infiltration (one (7.69%)), and fibrosis (one (7.69%)). 

The most common complication observed among patients in this study was mild cognitive impairment, accounting for two (15.38%) (Table 12).

**Table 12:** Complications of the cases involved in the study, with a total of 13 patients, expressed as a percentage (%)

Sr. no.	Complications	Number of cases, n (N = 13)	Percentage occurrence (n/N)
1.	Mild cognitive impairment	2	15.38%
2.	Sepsis	1	7.69%
3.	Left hemiplegia	1	7.69%
4.	Ventriculitis	1	7.69%
5.	Brain stem herniation	1	7.69%
6.	Respiratory failure	1	7.69%
7.	Metabolic encephalopathy	1	7.69%

Most common modes of treatment were combined (surgical and medical), in nine (69.23%) of patients, and medical in four (30.77%) of patients. Among empirical medical treatments, the drugs used were metronidazole (six (46.15%)), vancomycin (five (38.46%)), and ceftriaxone (four (30.76%)). No empirical treatment was given in 28.57%. On the other hand, the drugs used in the definitive treatment were metronidazole (eight (61.53%)), ceftriaxone (three (23.07%)), penicillin (three (23.07%)), and steroids (three (23.07%)) (Tables 13-14).

**Table 13:** Empirical treatment of the cases involved in the study, with a total of 13 patients, expressed as a percentage (%)

Sr. no.	Empirical treatment	Number of cases, n (N=13)	Percentage occurrence (n/N)
1.	Metronidazole	6	46.15%
2.	Vancomycin	5	38.46%
3.	Ceftriaxone	4	30.76%
4.	Meropenem	3	23.07%
5.	Gentamicin	1	7.69%
6.	Linezolid	1	7.69%
7.	Ceftazidime	1	7.69%
8.	Cefepime	1	7.69%
9.	Flucloxacillin	1	7.69%
10.	Trimethoprim-sulfamethoxazole	1	7.69%

**Table 14:** Definitive treatment of the cases involved in the study, with a total of 13 patients, expressed as a percentage (%)

Sr. no.	Drugs used in definitive treatment	Number of cases, n (N = 13)	Percentage occurrence (n/N)
1.	Metronidazole	8	61.53%
2.	Ceftriaxone	3	23.07%
3.	Penicillin	3	23.07%
4.	Steroid	3	23.07%
5.	Piperacillin-tazobactam	2	15.38%
6.	Anti-convulsant	2	15.38%
7.	Fosfomycin	2	15.38%
8.	Meropenem	2	15.38%
9.	Amoxicillin/clavulanic acid	1	7.69%
10.	Ampicillin/sulbactam	1	7.69%
11.	Clindamycin	1	7.69%
12.	Vancomycin	1	7.69%
13.	Flucloxacillin	1	7.69%
14.	Ceftazidime	1	7.69%

The most common surgical intervention used to treat brain abscesses in these studies was craniotomy (three (23.07%) and extraventricular drain placement (three (23.07%). Drainage of the abscess by any means led to the clinical improvement (Table 15).

**Table 15:** Surgical intervention used to treat brain abscess Fusobacterium nucleatum: occurrence and percentage, where N = 13

Sr. no.	Intervention/surgery	Number of cases, n (N = 13)	Percentage occurrence (n/N)
1.	Craniotomy	3	23.07%
2.	Extra ventricular drain placement	3	23.07%
3.	Robot-assisted guidance puncture biopsy	2	15.38%
4.	Neurosurgical stereotactic aspiration and drainage	1	7.69%

After management, 10 (76.92%) of patients were improved with definitive treatment (the antimicrobial therapy given based on culture or PCR findings). MRI done in four patients at an average of 23.4 weeks showed complete resolution in two (15.38%) patients and a reduction in abscess cavity size in the other two (15.38%) patients. The percentage of patients improved, remained static, and showed deterioration with empirical treatment (the initial broad-spectrum antimicrobial therapy administered before organism identification) remained the same at three (23.07%). Nearly three (23.07%) of patients died, and two (15.38%) deteriorated on definitive treatment (Table 16).

**Table 16:** Patient outcomes of the cases involved in the study, expressed as a percentage (%)

Sr. no.	Clinical status of patients	Number of studies, n (N = 13)	Percentage
1.	Improved with definitive treatment	10	76.92%
2.	Improved with empirical treatment	3	23.07%
3.	Static on empirical treatment	3	23.07%
4.	Deteriorated on empirical treatment	3	23.07%
5.	Death	3	23.07%
6.	Deteriorated on definitive treatment	2	15.38%

The details of the included studies are illustrated in the table below (Table 17).

**Table 17:** Details of included studies

Author	Study title	Year of publication	Country of publication	Study design
Zhang et al. [7]	Case report: two case reports of cryptogenic brain abscess caused by *Fusobacterium nucleatum* and literature review	2023	United States	Case report
Srour et al. [8]	Ruptured intraventricular brain abscesses due to *Fusobacterium nucleatum* with obstructive hydrocephalus: illustrative case	2023	United States	Case lessons
Laarif et al. [9]	Actinomyces israelii and *Fusobacterium nucleatum* brain abscess in an immunocompetent patient: case report	2023	Belgium	Case report
Toumeh et al. [10]	Fatal case of liver and brain abscesses due to *Fusobacterium nucleatum*	2021	United States	Case report
Chen et al. [11]	*Fusobacterium nucleatum*-caused brain abscess - case report	2021	Taiwan	Case report
Franceschi et al. [12]	Brain abscess and periodontal pathogens (*Fusobacterium nucleatum*). Report of a case	2020	Italy	Case report
Chakvetadze et al. [13]	Detection of *Fusobacterium nucleatum* in culture-negative brain abscess by broad-spectrum bacterial 16S rRNA Gene PCR	2017	France	Case report
Dhaya et al. [14]	*Fusobacterium nucleatum* endocarditis presenting as liver and brain abscesses in an immunocompetent patient	2015	United States	Case letter
Nagalingam et al. [15]	Identification of occult *Fusobacterium nucleatum* central nervous system infection by use of PCR-electrospray ionization mass spectrometry	2014	United states	Case report
Hischebeth et al. [16]	Rapid brain death caused by a cerebellar abscess with *Fusobacterium nucleatum* in a young man with drug abuse: a case report	2014	Germany	Case report
Kai et al. [17]	A rare presentation of ventriculitis and brain abscess caused by* Fusobacterium nucleatum*	2008	United Kingdom	Case report
Heckmann et al. [18]	Multiple brain abscesses caused by Fusobacterium nucleatum treated conservatively	2003	Germany	Case report

Discussion

Bacterial etiology underlines a substantial majority - approximately 85% of the spontaneous occurrence of brain abscess - with a significant proportion being attributed to dental and oropharyngeal microbiota [9]. Moreover, the polymicrobial nature of 30 to 60% of brain abscesses complicates the accurate determination of the primary infectious agent. Notably, anaerobic bacteria exhibit a threefold higher association with brain abscesses compared to aerobic bacteria [9]. *F. nucleatum* is a gram-negative anaerobic bacterium, commonly found in the oral, gastrointestinal, respiratory tracts, and genital tracts. The pathogen’s ability to invade host tissue, induce inflammation, and capacity to thrive in an anaerobic environment while enhancing the infectivity of other pathogens play a vital role in its pathogenicity [19].

The mean age in patients with *F. nucleatum* brain abscess was 48 ± 16 years compared to a study by Widdrington et al. and Lange et al., where the median age in patients with brain abscesses is 58 and 53 years, respectively, predominantly occurring in males [1,20].

The clinical presentation in patients with brain abscesses can vary based on location, size, and presence of underlying complications such as ventriculitis and obstructive hydrocephalus [8]. In this study, patients with brain abscesses presented with varying symptoms, that is, headache in seven (53.85%), fever in five (38.46%), and unilateral limb weakness in four (30.77%) patients. Compared to the study by Widdrington et al., where a retrospective analysis of 113 patients reported focal neurological deficits in 28% of patients and a reduced Glasgow Coma Scale in 39% of patients. [1]. Focal neurological symptoms rely on the affected site of the lesion [7]. The majority of lesions in this review were present in the frontal and cerebellar lobes, four (30.76%) each, with the majority of lesions located on the left side, six (46.15%). The average size of the lesion was 2.15 ± 0.91 cm. The size of the abscess can be used to assess the treatment approach, as Chen et al. suggested that medical therapy alone is suitable for abscesses smaller than 2.5 cm, in patients with GCS >12 with microorganisms being identified on sources other than abscess pus [21]. However, if medical therapy fails after one to two weeks, surgery should be considered. For multiple abscesses, medical treatment alone may suffice once abscesses are larger than 2.5 cm or those causing significant mass effort have been removed [21].

In our review, poor dental health or periodontitis was present in seven (53.85%) of patients and may point toward an association with brain abscesses due to *F. nucleatum*, consistent with its abundant presence in dental plaque and potential for hematogenous dissemination established in literature [22]. This signifies that oral examination in these patients is important, and routine oral hygiene should be maintained, as it is the most important risk factor in our study. The presence of extracranial *F. nucleatum* infections, noted in four (30.76%) patients (including liver and pulmonary abscesses), may also represent a risk factor, although causality cannot be firmly established. Extracranial sources in our study included liver and pulmonary abscesses, which is in parallel to a study by Bourgault et al., highlighting various extracranial sites involved in abscess formation by *F. nucleatum*, including skin, pleura, and liver [23]. Out of three patients with liver abscesses, two patients yielded growth of *F. nucleatum*. Interestingly, both patients deteriorated after aspiration and drainage of the liver abscess. According to the literature, vascular damage and atherosclerosis due to smoking and hypertension facilitate bacterial adherence and systemic dissemination and predispose individuals to *F. nucleatum* infections (hematological disseminations from the oral cavity) [4]. In our study, other risk factors included alcohol consumption in four (30.76%), smoking in three (23.07%), hypertension in two (15.38%), and endocardial vegetation in two (15.38%), predisposing patients to high levels of *F. nucleatum*. Presence of *F. nucleatum* has been seen in atherosclerotic plaques.

It is observed that six (69.23%) of patients were evaluated by MRI and seven (53.85%) by CT scan. Literature suggested that MRI, when combined with diffusion-weighted imaging (DWI) and apparent diffusion coefficient (ADC) imaging, is effective for distinguishing brain abscesses from primary, cystic, or necrotic tumors [24]. Key indicators of brain abscess include high-intensity signals with peripheral low-intensity rims, surrounded by an area of edema with a high-intensity signal on T2-weighted imaging, rim enhancement on contrast-enhanced T1-weighted imaging, and specific signal patterns on DWI and ADC imaging within the lesion center [4]. Further radiological investigation included echocardiography, which was done in two (15.38%) patients to evaluate potential cardiac sources of abscess.

In our study, the modalities used for the detection of *F. nucleatum* were nucleic amplification techniques in nine (69.23%), abscess culture in six (69.23%), and gram staining in two (15.38%). Alon-Maimon et al. suggested that the most predominant tool utilized for the quantification of *F. nucleatum* is quantitative PCR [22]. However, PCR is not routinely performed [16] in most of the centers, likely due to cost constraints and limited availability of molecular diagnostic facilities in many institutions. Heckmann et al. also highlighted the use of nucleic acid-based detection methods, particularly when empirical antibiotics may inhibit culture growth, it identified the presence of *F. nucleatumin* CSF using 16S ribosomal RNA (rRNA) [18]. Among samples of brain abscess and CSF sent for PCR, the former gave more positive results, in five (38.46%).

Cultures were performed on blood samples, CSF, and aspirated brain abscess samples. Among all samples, it is observed that abscess culture yielded the most positive results in four (30.77%), in contrast to the majority of blood in eight (61.53%), and CSF in six (46.15%), which showed no growth. These findings are, however, in contrast with a study by Lange et al., suggesting positive blood cultures in 29% of patients with brain abscess [20]. The low yield of blood and CSF culture could be due to empirical antibiotic therapy before sampling, difference in microbiological techniques. Furthermore, the strict anaerobic growth requirements and sensitivity to handling make isolation of *F. nucleatum* difficult as well.

Given the rising incidence of brain abscesses, it is important to employ evidence-based therapy to ensure effective treatment [20]. In this study, the majority of patients were managed both surgically and medically in nine (69.23%). Antimicrobial therapy plays a pivotal role [21]. The preferred antibiotic should penetrate the blood-brain barrier and exhibit bactericidal properties. Upon suspecting brain abscess, it is recommended to commence empirical broad-spectrum antibiotic encompassing coverage of both gram-positive and anaerobic organisms with agents like a third-generation cephalosporin combined with metronidazole [21]. In this review, empirical antibiotic therapy included metronidazole in six (46.15%), vancomycin in five (38.46%), ceftriaxone in four (30.76%), and meropenem in three (23.07%) patients. It was similar to the study by Zhang et al., which stated that the use of third-generation cephalosporins in combination with metronidazole was used in immunocompetent patients. However, in patients with a history of neurosurgical procedures, vancomycin could be added, and meropenem can be used as an alternative to ceftriaxone or where metronidazole is contraindicated [7].

Once *F. nucleatum* is known to be the causative organism, pharmacological agents were narrowed down to metronidazole in eight (61.53%), ceftriaxone in three (23.07%), penicillin in three (23.07%), and steroids in three (23.07%). Metronidazole 500 mg thrice daily was given for an average of 13 weeks, and ceftriaxone 2 g once daily was given for an average of six weeks. The use of metronidazole as a priority antibiotic aligns with the literature, which suggests that metronidazole attenuates the pro-tumorigenic effects of *F. nucleatum*, decreases the bacterial load, and has a high sensitivity for anaerobes [1,19,22]. A study similar to that by Dhaya et al. suggested that penicillin G and metronidazole are an effective treatment for fusobacterium septicemia [14]. The average hospital stay was observed to be 17.33 days.

Surgery plays an important role in relieving intracranial pressure symptoms and collecting abscess samples to identify causative organisms [7]. However, this approach is in contrast to a study by Chen et al., stating that potential risk and morbidity associated with brain abscesses, such as cerebral oedema, hemorrhagic complications, and risk of dissemination due to pus leakage can be avoided by withholding surgery [11]. Increasingly, the literature supports stereotactic aspiration coupled with systemic antibiotics as a preferred surgical approach, which can be safely executed and can yield positive culture results [21]. It is observed that surgical procedures used in patients were craniotomy in three (23.07%), extraventricular drain placement in three (23.07%), assisted guidance puncture biopsy in two (15.38%), and neurosurgical stereotactic aspiration and drainage in one (7.69%). Overall, 10 (76.92%) of patients showed improvement with definitive treatment (surgical and medical), with 50% of patients among those showing complete resolution of abscess on MRI with an average of three months of follow-up. However, two (15.38%) showed deterioration with complications such as ventriculitis, sepsis, and respiratory failure. One patient had a medical history of hypertension, and the other had an extracranial source of infection (liver abscess); both patients also had poor dental hygiene. The observed complications were most likely secondary to the underlying brain abscess caused by *F. nucleatum*.

Limitations

The findings in the study should be interpreted cautiously, considering the small sample size, retrospective nature of reports, and heterogeneous case sources. Furthermore, certain case reports suggest suboptimal clinical outcomes in treating infections caused by *F. nucleatum*, underscoring the complexity in identifying definitive treatment. Additionally, we limited our study to the PubMed search engine. This was done to avoid duplication and for the credibility of the selected articles.

Clinical Recommendations

Further clinical investigation is needed to firmly establish *F. nucleatum* as one of the etiological factors for various carcinomas. Retrospective clinical research studies with substantial data size are crucial to establish the transmission routes of *F. nucleatum* and identify factors predisposing patients to *F. nucleatum*-associated abscesses. The implementation of clinical trials is warranted to formulate effective management strategies for diverse abscesses caused by this microorganism.

As per the detailed literature review we have conducted (Table 17), we propose a flowchart for the management of cases of *F. nucleatum* brain abscess (Appendix A and Appendix B).

## Conclusions

This study highlights the importance of recognizing potential risk factors for *F. nucleatum* brain abscesses, particularly poor oral hygiene. Given the high rate of negative blood and abscess cultures, molecular diagnostics such as NAAT/PCR could be valuable tools for culture-negative cases. Furthermore, effective management often requires a combination of appropriate antibiotics, such as metronidazole, penicillin, and adjunctive steroids, with surgical drainage. This study also signifies thorough oral examination should be considered an essential component of the assessment in affected patients.

## Figures and Tables

**Figure 1:**
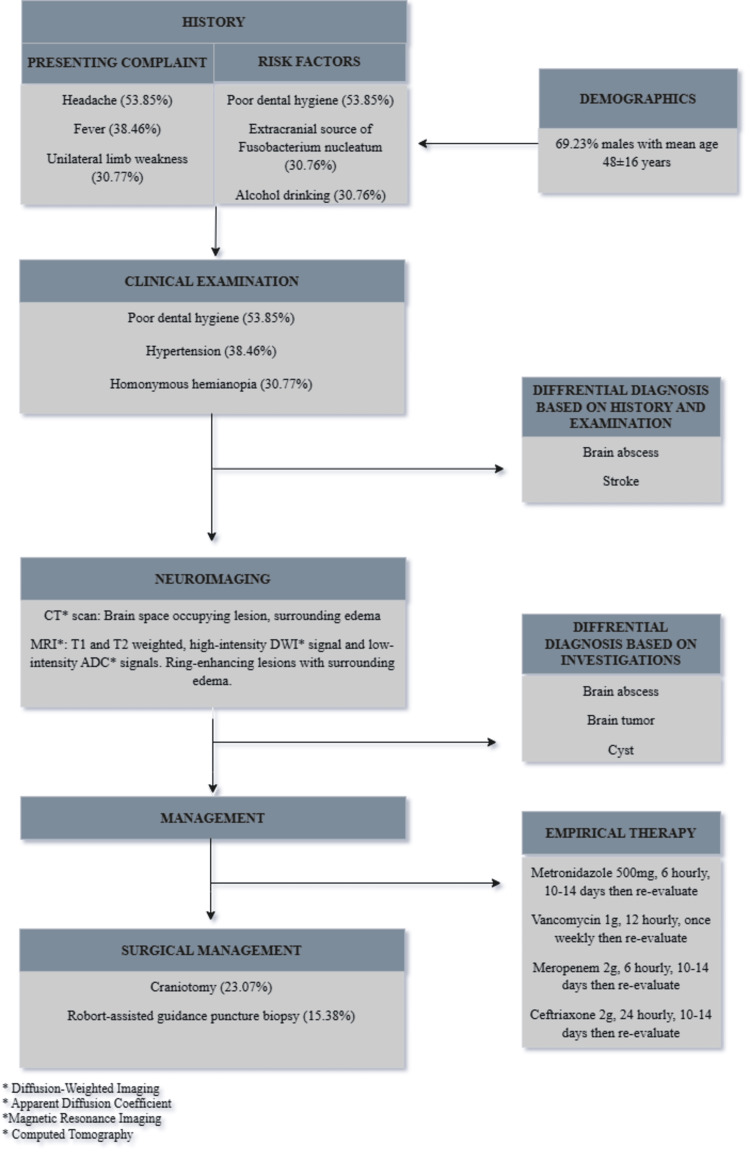
Author-proposed illustration of management of cases of Fusobacterium nucleatum brain abscesses of included cases

**Figure 2:**
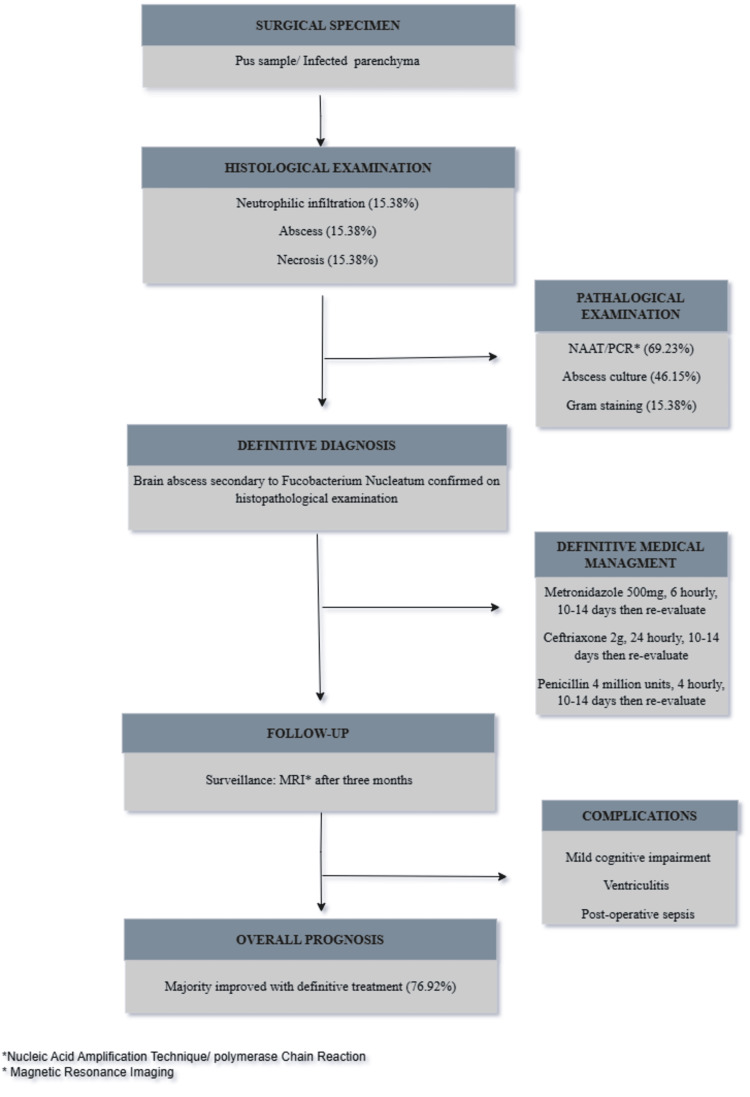
Continuation of the author-proposed illustration of management of cases of Fusobacterium nucleatum brain abscess of included cases

## Appendices

Appendix A 

Appendix B 
